# Bowel obstruction and perforation secondary to barbed suture after minimally invasive inguinal hernia repair: report of two cases and literature review

**DOI:** 10.1186/s40792-021-01249-w

**Published:** 2021-07-13

**Authors:** Liming Wang, Taku Maejima, Susumu Fukahori, Shoji Nishihara, Daitaro Yoshikawa, Toru Kono

**Affiliations:** Department of Surgery, Sapporo Higashi Tokushukai Hospital, 3-1, N-33, E-14, Higahi-ku, Sapporo, Hokkaido 0650033 Japan

**Keywords:** Barbed suture, Complications, TAPP

## Abstract

**Background:**

Laparoscopic transabdominal preperitoneal patch (TAPP) is now commonly used in the repair of inguinal hernia. Barbed suture can be a fast and effective method of peritoneal closure. We report two rare cases of small bowel obstruction and perforation caused by barbed suture after TAPP.

**Cases:**

Patient 1 is a 45-year-old man who underwent laparoscopic repair of a right inguinal hernia. Barbed suture was used to close the peritoneal defect. At 47 days after the operation, he was diagnosed with a small bowel obstruction caused by an elongated tail of the barbed suture. Emergency laparoscopic exploration was performed for removal of the embedded suture and detorsion of the volvulus. The second patient is a 50-year-old man who was admitted with a small bowel perforation one week after TAPP herniorrhaphy. Emergency exploration revealed that the tail of the barbed suture had pierced the small intestine, causing a tiny perforation. After cutting and releasing the redundant tail of the barbed suture, the serosal and muscular defect was closed with 2 absorbable single-knot sutures. Both patients have recovered well. Finally, we searched the PubMed database and reviewed the literature on the effectiveness and safety of barbed suture for TAPP.

**Conclusions:**

Surgeons should understand the characteristics of barbed suture and master the technique of peritoneum closure during TAPP in order to reduce the risk of bowel obstruction and perforation.

## Background

Laparoscopic transabdominal preperitoneal patch (TAPP) is now commonly used in the repair of inguinal hernias [1, 2]. In order to avoid adhesions between the bowel and the patch and to prevent bowel obstruction due to herniation into the preperitoneal space [3, 4], the peritoneum must be closed continuously and completely after the patch is placed.

Barbed suture is a unidirectionally barbed, self-anchored, non-slip suture that is now widely used in skin repair [5, 6], digestive tract reconstruction, and obstetrics and gynecology [7–9]. Barbed suture is also favored for laparoscopic hernia repair because there is no need to tie knots at the end of the suture. These barbs can also be fixed in the peritoneum to firmly repair the peritoneal defect [10].

With the introduction of new technologies, complications will eventually arise. Here, we present two cases of bowel obstruction and perforation after TAPP repair, which were both related to the barbed suture. We also review the recent literature to provide some objective suggestions for the future.

## Case presentation

### Case 1: bowel strangulation caused by barbed suture

**Fig. 1:**
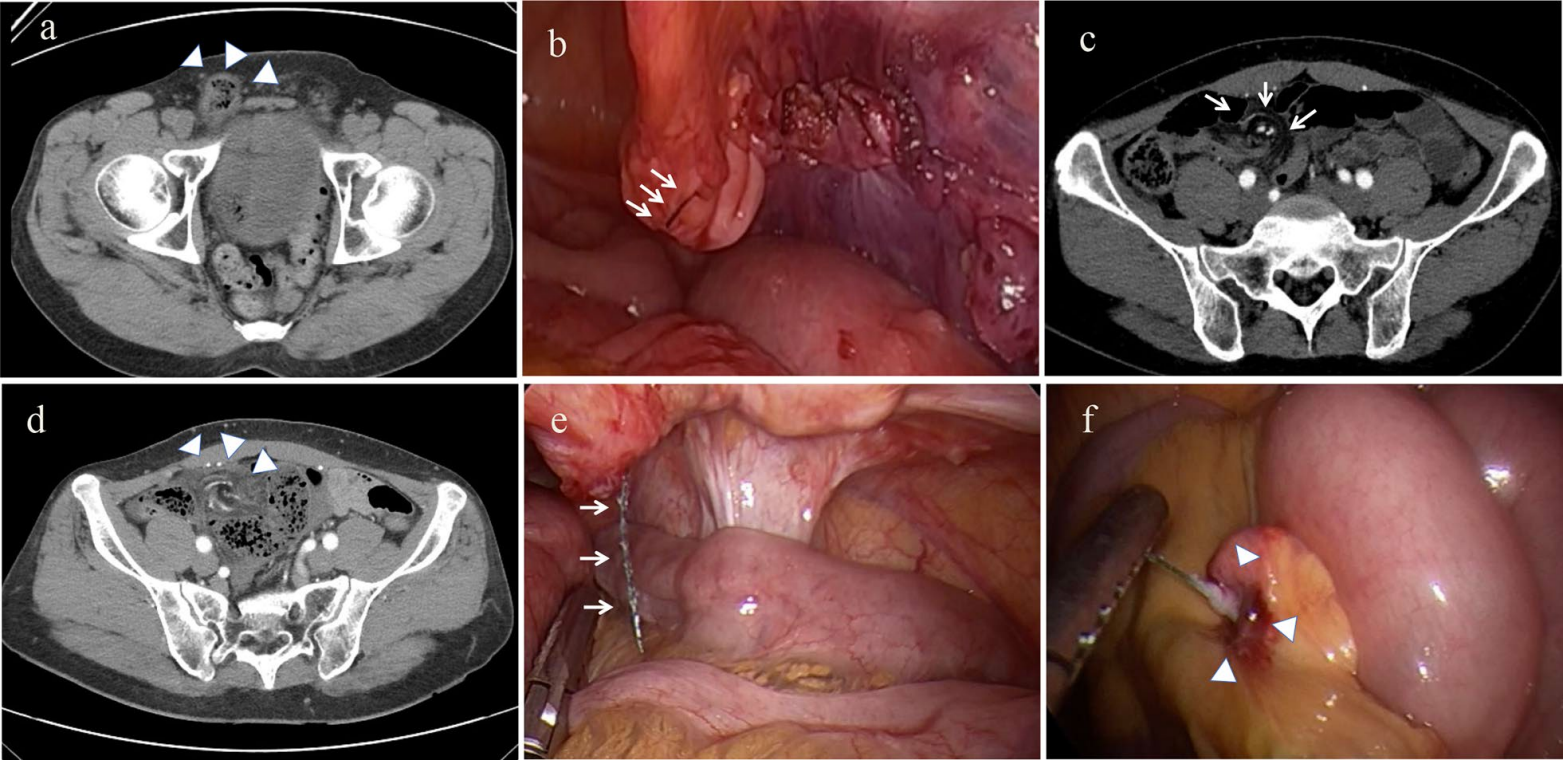
Preoperative and intraoperative images in Case 1. **a** CT shows right indirect inguinal hernia in prone position (white arrowheads). **b** A self-anchoring barbed suture device is used to close the peritoneal defect from the right side to the left. The residual tail is about 5 mm (white arrows). **c** Upon readmission due to vomiting on postoperative day 3, the CT scan shows a small bowel obstruction with possible volvulus of ileum (white arrows). **d** A small bowel obstruction with strangulation one month later (white arrowheads). **e** The tail of the barbed suture is much longer and the bowel segment is strangulated (white arrows). **f** The tail of barbed suture attached to the mesentery of the distal ileum (white arrowheads)

A 45-year-old man with swelling in the right groin due to indirect hernia (Fig. 1a). TAPP repair was performed by an experienced surgeon and the peritoneal defect was closed with a 4-0 absorbable monofilament barbed suture (V-loc™, Covidien, Mansfield, MA, USA) from right to left, leaving a residual tail of about 5 mm (Fig. 1b).

The patient was discharged on the first day after surgery but was readmitted on the following day because of abdominal pain and vomiting. The CT scan at readmission showed a small bowel obstruction with possible ileal volvulus (Fig. 1c). Although the symptoms resolved spontaneously after 2 days of fasting and rehydration, the patient continued to have intermittent abdominal pain and he was readmitted again on postoperative day 47 with worsening abdominal pain. The CT showed dilated small bowel with the mesentery rotating around the mesenteric vessels, which prompted suspicion of small bowel obstruction with strangulation (Fig. 1d). Emergency laparoscopic exploration revealed that the tail of the barbed suture was much longer and was embedded in the small bowel mesentery, causing volvulus obstruction (Fig. 1e, f). We cut the residual end of the barbed suture and removed the embedded tail of the barbed suture from the mesentery. The patient was discharged on the fourth postoperative day.

### Case 2: perforation of the small intestine due to the barbed suture

**Fig. 2:**
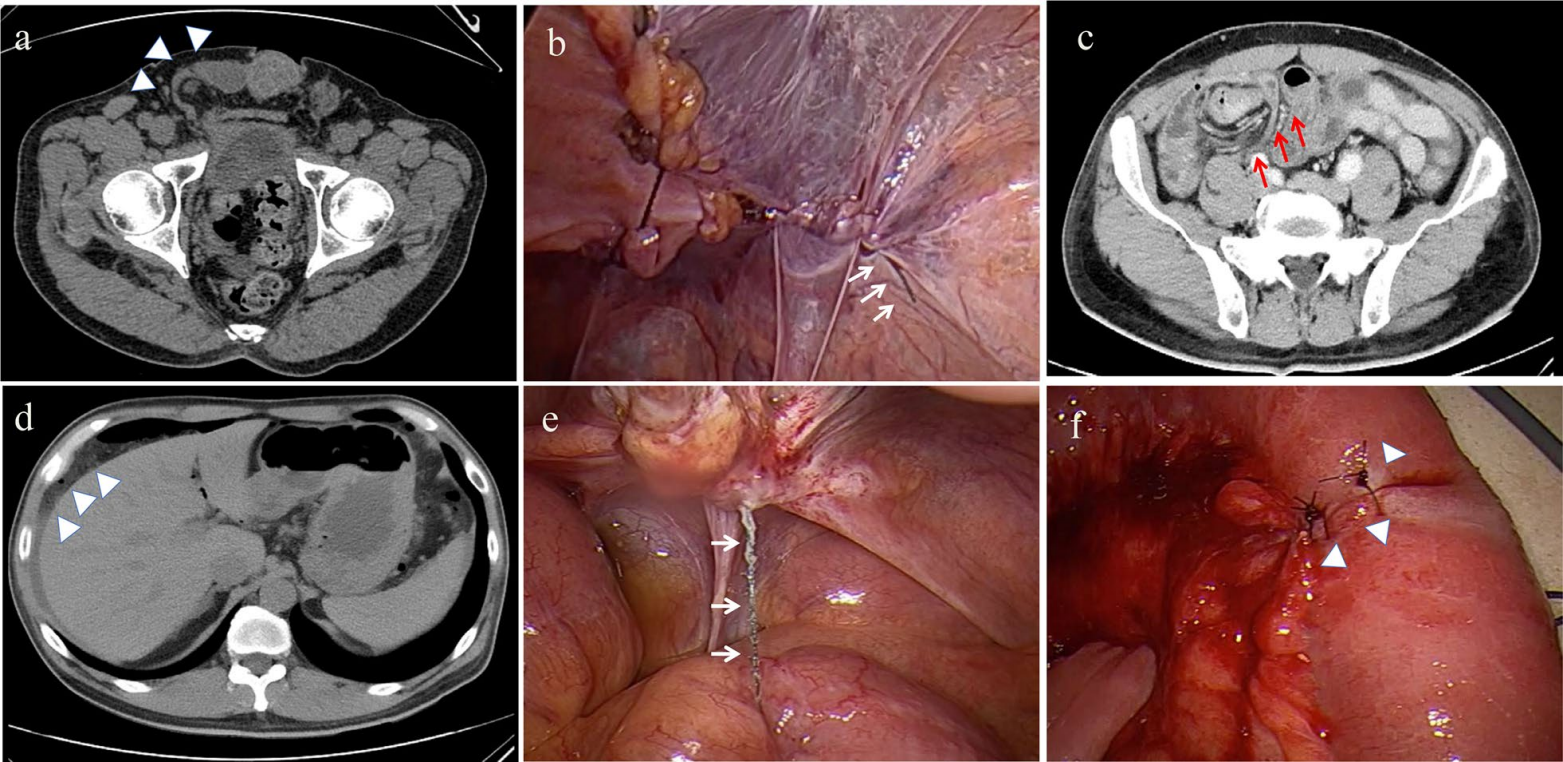
Preoperative and intraoperative images in Case 2. **a** The CT shows a right direct inguinal hernia in the prone position (white arrowheads). **b** The self-anchoring barbed suture device is used to close the right inguinal peritoneal defect from the left side to the right. The residual tail is less than 1 cm (white arrows). **c** The patient was readmitted with unremitting abdominal pain on postoperative day 10. There is obvious edema of small intestine, possibly volvulus of the ileum (red arrows). **d** Free air and ascites in the abdominal cavity, indicating perforation of the small intestine (white arrowheads). **e** The tail of the barbed suture is much longer and has inserted into the small intestine (white arrows). **f** After release of redundant barbed suture and removal of the tail of the barbed suture the perforated small intestine is closed with 2 stitches (white arrowheads)

A 50-year-old man presented with a preoperative CT diagnosis of a right direct inguinal hernia (Fig. 2a). TAPP repair was performed by a trainee surgeon, and the peritoneal defect was closed from left to right with V-loc™ (Covidien, Mansfield, MA, USA). The residual tail was < 1 cm (Fig. 2b). At 1 week after surgery, the patient developed unremitting abdominal pain, and the symptoms continued to worsen. On the 10th day, the CT scan showed obvious edema of the small intestine, which was possible ileal volvulus (Fig. 2c). There was free air as well as ascites in the abdominal cavity, indicating bowel perforation (Fig. 2d). At emergency laparoscopic exploration, we found that the elongated tail of the barbed suture had pierced into the small intestine (Fig. 2e). After cutting and releasing the redundant barbed suture and removing the tail of the barbed suture, the serosal and muscular defect was closed with 2 absorbable single-knot sutures. The patient was discharged from the hospital on the 7th postoperative day. Both patients have recovered well.

## Discussion

TAPP is generally very safe and minimally invasive [11]. However, postoperative intestinal obstruction occurs occasionally, usually because of adhesions between the bowel and the mesh or due to an internal hernia through the peritoneal defects [3, 10]. Therefore, the exact closure of peritoneal defects is an indispensable procedure of TAPP surgery. Traditional peritoneal closure methods include tacks, clips, staples, strap devices, etc. [4], but these may damage the nerves of the abdominal wall and increase the risk of postoperative chronic pain. The placement of running sutures for closing the peritoneal defect is one of the surgical techniques that must be mastered in TAPP [12]. However, intra-abdominal suturing is time-consuming and technically demanding.

The barbed suture is self-anchored and there is no need for knots. It promises an innovative technology for closing an incision faster without compromising strength and safety. This revolutionary product is now widely used in minimally invasive surgery [13, 14]. But new products cannot always be perfect. Recently, reports of related complications have been published [4, 15–22].

We searched on PubMed with keywords including barbed suture, complication, and TAPP, and found a total of eight case reports from 2012 to 2021. The onset time varies from 1 day to 3 months after surgery [18, 21, 22]. The main manifestations are abdominal pain and vomiting, and the later onset cases mainly manifest as uninterrupted abdominal pain or abdominal distension with worsening symptoms [17]. In the first case we experienced, the patient presented with unexplained vomiting and abdominal pain on the second day after surgery. However, due to lack of experience, we did not expect intestinal obstruction caused by the barbed suture. In a total of 9 cases in the literature, a tail of barbed suture has been seen penetrating into the mesentery, causing intestinal torsion, and ultimately leading to bowel obstruction. At press, none of the patients had lasting damage to the intestine (Table 1).

**Table 1:** Review of cases of complications caused by barbed suture after laparoscopic hernia repair

No.	Year, patients (*n*)	References	POD	Symptoms	CT finding	Intraoperative finding	Treatment
1	(2012) *n* = 1	Buchs et al. [18]	8 days	Diffuse abdominal pain	Small bowel obstruction with strangulation	Barbed suture in strangulated bowel segment	Division of barbed suture and bowel release
2	(2015) *n* = 1	Filser et al. [15]	3 days	Abdominal pain	Small bowel obstruction	Small bowel and mesenteric vessels twisted around barbed suture wire	Lysis of adhesions and removal of suture wire
3	(2015) *n* = 1	Köhler et al. [4]	13 days	Abdominal pain and vomiting	Piano key phenomenon (US)	The barbs were inseparably ingrown into the small bowel	Cut the barbed suture. Serosal and muscular defect was closed
4	(2018) *n* = 1	Tagliaferri et al. [19]	1 day	Abdominal pain and distension, vomiting	Small bowel volvulus	Small bowel volvulus with associated ischemia	Release of adherent suture and volvulus detorsion
5	(2019) *n* = 1	Sartori et al. [20]	3 days	Abdominal pain and vomiting	Small bowel obstruction	Abdominal adhesions and intestinal obstruction	Cut wire cut and release small bowel
6	(2020) *n* = 1	Zipple et al. [21]	1 day	Abdominal pain, vomiting, and mild leukocytosis	Significant dilated proximal small bowel	Terminal ileum was trapped	Cut and remove suture tail and release bowel
7	(2021) *n* = 1	Man et al. [17]	90 days	Recurring abdominal pain aggravated	The whirlpool sign of the mesentery	Intestinal volvulus	Lysis of adhesions and reduction of intestinal volvulus
8	(2021) *n* = 1	Zheng et al. [22]	3 days	Severe abdominal pain	Small intestinal dilation and volvulus	Small bowel obstruction and edema	Cutting the barbed suture and volvulus detorsion
9	(2021) *n* = 2	Our case 1	47 days	Recurring abdominal pain aggravated	Small bowel dilated with the mesentery rotating	Tail of the barbed suture inserted into the small bowel mesentery; intestinal obstruction	Removed the embedded barbed suture in the mesentery
		Our case 2	10 days	Uninterrupted abdominal pain aggravated	Free air and ascites in the abdominal cavity	The tail of the barbed suture punctured the small intestine	The serosal and muscular defect was closed with absorbable sutures

Reports of intestinal perforation following hernia repair are rare. Necrotic perforation has been reported in tack fixation [23]. In our second patient, the suture tail had penetrated directly into the bowel wall, resulting in a tiny perforation, which was closed with a simple suture of 2 stitches, and the mesh patch was not infected.

To investigate further, we reviewed the operation videos and discovered that the tail stump was actually < 1 cm during the first operation, but at the second operation the tail stump was 4–5 cm long. Similar findings have been reported in other cases [15, 18], and even if the tail was in accordance with the instructions, the barbed suture would still extend automatically. This result has also been well documented during in vitro animal experiments [16]. It is possible that as the peritoneum contracts during healing the barbed suture tail is squeezed out unidirectionally [16]. Once the tail of the barbed suture penetrates the mesentery or the bowel wall, the unidirectional anchoring characteristic would further increase the length of the involved suture as intestinal peristalsis increases.

Avoiding this complication is something that surgeons need to take seriously. Some experts suggest that the barbed suture tail needs to be sutured back with two stitches, so that the barbed suture is completely self-anchored [24]. However, animal in vitro experiments have shown that in addition to the free tail of the barbed suture, the barb itself could also hook the bowel [24]. Therefore, some surgeons routinely place anti-adhesion agents at the barbed suture site in the Japanese literature.

Even when the barbed suture is cut short enough as recommended [16], bowel obstruction or perforation can still occur in TAPP. Eliminating this risk completely remains a challenge for surgeons. We have summarized some precautions that should be considered when using barbed sutures: (1) the surgeon can appropriately reduce the pneumoperitoneum pressure when closing the peritoneal defect, thereby reducing the risk of peritoneal tears; (2) the surgeon can tighten the tail as much as possible so that the peritoneal folds can cover the barbs that are exposed to the abdominal cavity; (3) placing two back stitches at the end of the barbed suture to make sure they anchor accurately; (4) shortening the free barbed tails and not exposing the stump of the tail to the abdominal cavity. Random trials are needed to verify the effectiveness of routine use of anti-adhesion agents.

## Conclusion

Surgeons should understand the characteristics of barbed suture and master the technique of peritoneum closure during TAPP in order to reduce the risk of bowel obstruction and perforation.

## Data Availability

The datasets supporting the conclusions of this article are included within the article and its additional files.

## Abbreviations

TAPP: Transabdominal preperitoneal patch
CT: Computed tomography
US: Ultrasonography
